# Measuring the hepatic venous pressure gradient in the upper digestive hemorrhages

**Published:** 2015

**Authors:** S Omer, O Zara, A Iftime, I Dina

**Affiliations:** * ”Carol Davila” University of Medicine and Pharmacy, Bucharest; Department of Gastroenterology, “Sf. Ioan” Hospital, Bucharest, Romania; ** Department of Cardiology, ”Sf. Ioan” Hospital, Bucharest, Romania; *** Department of Biophysics, ”Carol Davila” University of Medicine and Pharmacy, Bucharest, Romania

**Keywords:** Hepatic venous pressure gradient, upper digestive hemorrhage, liver cirrhosis

## Abstract

The upper digestive hemorrhage is one of the main causes of mortality from liver cirrhosis (CH). The measurement of the hepatic venous pressure gradient (HVPG) by angiographic way can be used for the determination of the risk of hemorrhage. The aim of this study is to verify the influence of the HVPG measurement upon the survival in patients with CH and upper digestive hemorrhage. A retrospective pilot study on 33 patients with upper digestive hemorrhage divided into two groups according to the therapeutic attitude followed, was carried out. One group was classically treated, with variceal band ligation, non-elective beta-blockers and in emergency Octreotide. The other group was treated depending on the value of HVPG. From the moment of the first episode of digestive hemorrhage, the survival period was in average of 8.1 months for the patients with viral etiology and for those with alcoholic etiology it was of 19.7 months. In patients treated after HVPG measurement, the average survival period was of 34.2 months and in patients “classically” treated, the average survival period was of 15.5 months (significant difference).

In conclusion, the measurement of HVPG allowed the selection of a high-risk group of patients. This permitted the making of a therapeutical decision with a significant prolongation of life in these patients.

## Introduction

The upper digestive hemorrhage is one of the main causes of mortality from liver cirrhosis. The rate of death from upper digestive hemorrhage in patients with liver cirrhosis is 15-20% [**1**-**4**]. The main source of bleeding is ruptured esophageal varices and portal hypertensive gastropathy. Although several studies have defined a number of factors associated with a poor outcome in acute variceal bleeding [**4**-**6**], in most centers, the patients are treated in the same way regardless of the likelihood of a good or a bad outcome [**7**]. The determination of the risk of hemorrhage either by varicose veins rupture, or caused by hypertensive portal gastropathy imposes the measurement of the hepatic venous pressure gradient (HVPG), that is, the difference in pressure between the portal and the systemic circulation. Previous studies have found that the measurement of the HVPG by angiographic way is at least as important as the survival prognosis scores (CHILD, CHILD-TURCOTT, MELD) [**5**,**8**,**9**]. Various methods of measuring the HVPG or the sinusoidal pressure have been tried either by the endoscopic way, or by non-invasive evaluations of the sinusoidal pressure (liver elastography, splenic elastography, Doppler examination of the portal circulation), without being able to find a satisfying correlation. As a result of this, the angiographic method remains, at least for now, the main way of measuring the HVPG and thus the risk of serious bleeding in liver cirrhosis.

Considering that there are already endoscopic signs of the risk of bleeding again, we asked ourselves what are the clinical benefits regarding the survival, through the measurement of the HVPG by the angiographic method, in patients with cirrhosis of the liver and digestive hemorrhage. Is it enough to measure this gradient or the current protocols mainly based on endoscopic, clinical and biohumoral features? It is known from the previous studies that the therapeutic failure rate was of 30-40% during the period when the therapy was based on endoscopic variceal sclerotherapy or on vasoactive medication [**5**,**6**,**9**], while, when the therapy was actually based on vasoactive medication, variceal band ligation and antibiotics, the failure rate has dropped to 15-20% [**1**-**3**,**10**,**11**].

**Aim of the study**

The study deals with the implications of the measurement of the hepatic venous pressure gradient (HVPG) with the consecutive therapeutic measures on the duration of the survival in patients with liver cirrhosis and upper digestive hemorrhage, hospitalized in the Medical Clinic of “Sf. Ioan” Hospital in the last 3 years.

## Material and method

Patients with liver cirrhosis, regardless of its etiology, hospitalized in the Medical Clinic of “Sf. Ioan” Hospital in the last 3 years, were monitored. Of these patients, overall 250, 40 deaths were found. Of these, 11 deaths were due to the upper digestive bleeding (29%) and to portal hypertension.

**Fig. 1:**
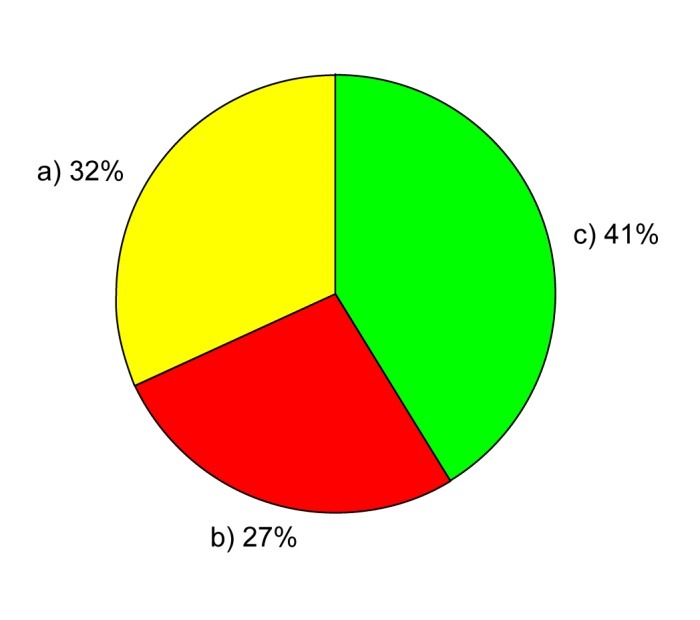
Mortality in liver cirrhosis patients observed in this study. a) Portal-induced encephalopathy, 32%; b) Hemorrhagic events in the upper digestive tract, 27%; c) Other etiologies, 41%

Hospitalized patients with upper digestive hemorrhage due to portal hypertension (ruptured esophageal varices, portal gastropathy) were selected and divided into two groups according to the therapeutic attitude approached. Basically, a retrospective pilot study on 33 patients with upper digestive hemorrhage, divided into two groups according to the therapeutic attitude, was carried out.

The group A consisted of 22 patients (9 women and 13 men) with digestive hemorrhage classically treated, with variceal band ligation, nonselective beta-blockers and in emergency Octreotide.

The group B consisted of 11 patients with digestive hemorrhage for whom HVPG was measured by the angiographic method. The treatment decision was made according to the value found. For 10 of them, for whom a gradient over 20mmHg was found, a decompression method of the port system was made.

On 7 patients, a transjugular portosystemic shunt (TIPS) was conducted, a splenorenal surgical shunt with splenectomy was performed on 2 patients and a liver transplant was performed on a patient. The 11th patient of group B had a HVPG under 16mmHg following the classical treatment with beta-blockers and variceal band ligation.

The period of survival from the first upper digestive hemorrhage with which they presented in our clinic, compared to the group A as reported to group B, was watched in the studied groups.

There was also an interest in finding out if the etiology or sex had any influence on the post hemorrhagic episode evolution in the patients of group A. Moreover, an interest was also manifested regarding the evolution of these patients because of the 11 deaths by upper digestive hemorrhage, 10 (90.9%) were from this group and only one death (9,1%) was from group B.

The duration (in months) was quantified from the first HDS episode until the death or until a fixed moment (April 2014). This period was called the “survival period”.

The periods registered on this group of patients were from 0 months (death on the first HDS episode) to 38 months (patient who was still under observation).

It was found that, as far as the distribution of the duration on the two sexes in group A were concerned, there was no significant difference.

If the repartition of the survival period was analyzed by etiological categories (viral cirrhosis, alcoholic cirrhosis, respectively) in group A, a significant difference between the two groups was observed, as it follows:

- 8 patients with viral etiology, the average survival period 8.1 months, standard deviation 8.0 months

- 14 patients with alcoholic etiology, the average survival period 19.7 months, standard deviation 9.6 months

With a 95% confidence, it can be stated that between the two types of etiologies, a significant difference of the survival period (ANOVA test, p-value = 0.012) was observed in the group of patients. The analysis of the distribution of the variation of the mediums and the distributions of the populations was done with the VisualStatistics v6.4 package.

Although the number of patients is small and the groups are unevenly distributed, these results can probably be extrapolated on broader populations of patients, and there is an intention of studying the impact of the various types of treatments on the survival period from the first HDS episode more thoroughly. What should be mentioned is the fact that the period in months was expressed due to an uncertainty regarding the accuracy of data in the history of 4 patients.

**Fig. 2:**
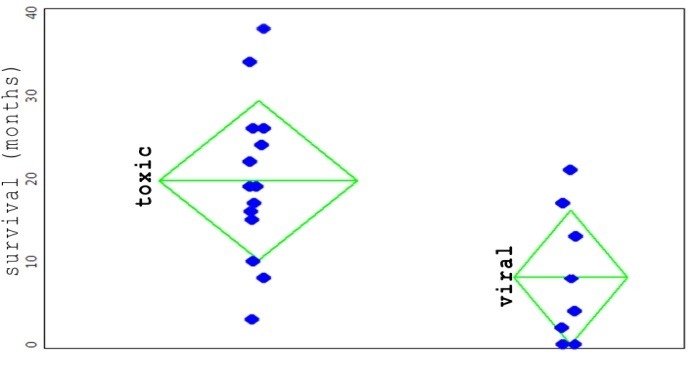
The distribution of patients according to etiology (the liver alcoholic etiology, respectively viral groups). Each blue dot represents a patient. Upright, the survival duration in months. In green, the average survival duration in that respective group. The tips of the triangle: the standard deviation. The average survival duration in the group with alcoholic etiology: 19.7 months; in the group with viral etiology: 8.1 months. The two groups are significantly different from the statistical point of view (see text).

**Fig. 3:**
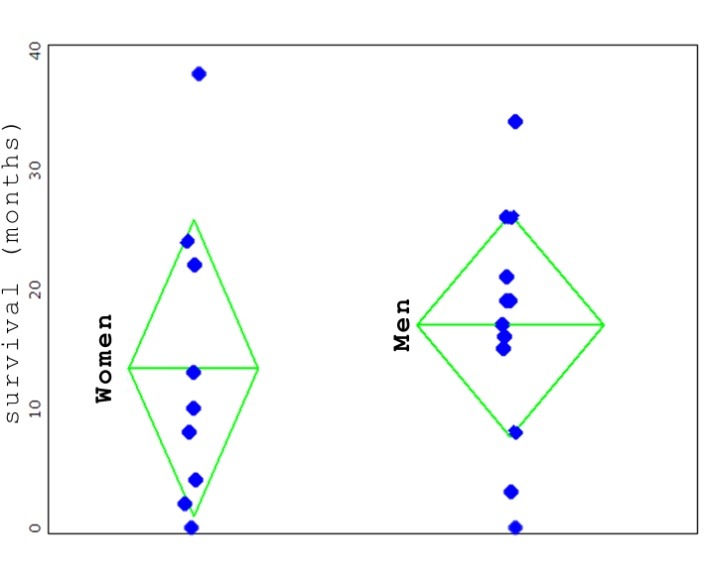
The distribution of the patients according to their sex. Each blue dot represents a patient. Upright, the survival period, in months. In green, the average of the survival period within that group; 13.4 months for women, 17 months for men. The difference between the averages is not statistically significant.

These positive results have encouraged us to try the retrospective assessment of the patients’ evolution (survival period) and depending on the treatment. Thus, besides the patients medically (“classical”) treated, the interventionally treated patients (surgical shunt, TIPS, 1 case of transplantation) were also evaluated.

The distribution was:

-22 patients “classically” treated, the average survival period of 15.5 months, standard deviation 10.5 months

-10 patients treated with an interventional “new” treatment, the average survival period 34.2 months, standard deviation 18.9 months.

It can be said that for the mentioned group, with a 95% confidence, the two groups were significantly different from the statistical point of view (ANOVA test, p-value < 0.0012). However, it should be noted that within the second group of patients, the level of confidence of the accuracy of the data was low (/-6 months) per patient. This was because the date of the first HDS was obtained at times from the history (through anamnesis) and not documented in the database of the hospital (non-existent at the time).

Even with this observation, the difference between the two groups was clinically important; to observe that those in the second group, also had the pressure gradient evaluated prior to the intervention (high values being found), which justified the intervention. From the data collected so far, it can be stated that an assessment of the gradient of pressure followed by a therapeutic intervention for its balancing, leads to a significantly longer survival period. As a final observation, the period of 34.2 months refers to patients who were included in this study and who are, the great majority of them, alive.

**Fig. 4:**
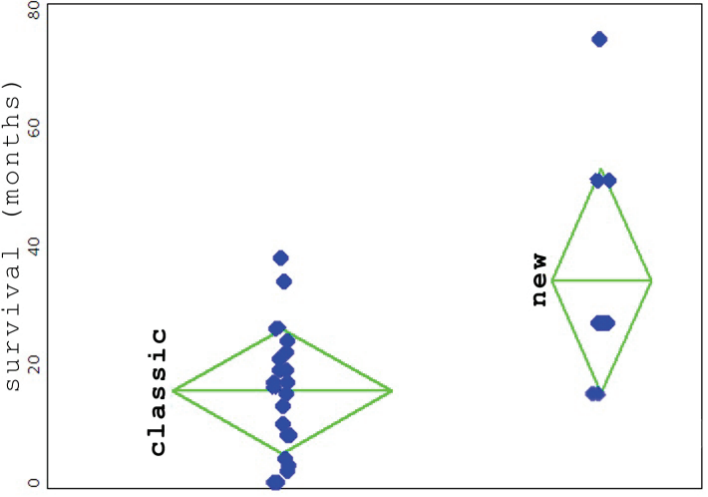
The distribution of the patients according to the treatment type after HDS (the group with classical treatment, respectively new interventional treatment of TIPS type, surgical shunt, transplantation). Each blue dot represents a patient. Upright, the survival period, in months. In green, the average length of the survival period within the respective group. The tips of the triangle: the standard deviation. The average length of the survival period within the group with classical treatment: 15.5 months; in the group with HVPG measurement: 34.2 months. The two groups are significantly different from statistical point of view (see text).

## Discussions

It can be affirmed that from the moment of the first episode of digestive hemorrhage on the studied groups, the survival period was in average of 8.1 months for the patients with viral liver cirrhosis etiology and for those with alcoholic etiology it was of 19.7 months. The difference in survival could be explained by the fact that in the patients with viral etiology, the so-called irreversible component of the portal high blood pressure given by the intra-hepatic fibrosis wasmore marked, while in patients with alcoholic etiology, the reversible component given by the vasomotor and neurohumoral alterations was more marked. As Monescillo and co-workers have shown [**9**], the measurement of early HVPG is the main determinant of treatment failure and death, and that early TIPS placement reduces treatment failure and mortality in patients qualified as high risk on hemodynamic criteria. At the present, we do not have the possibility of identifying the differences between the two components of portal hypertension than by indirectly measuring the Doppler intra-portal flow or of the HVPG before and after the beta-blockers measurement, which is impossible to achieve in emergency situations. A HVPG ≥20mmHg was shown to predict failure of medical and endoscopic treatment in acute variceal bleeding [**5**]. In these patients, it was decided that a method for lowering the portal pressure (TIPS, surgical shunt, liver transplant) should be applied. As a result, their survival rates were higher (34.2 vs 15.5 months). So, it can be appreciated that in these patients, with an over 20mmHg gradient (basically with the imminence of a variceal rupture), the irreversible component of the portal hypertension, meaning that component uninfluenced by beta-blockers or nitrates, was so high, that, without a reduction of the pressure within the portal system (TIPS, surgical shunts, liver transplant), the life expectancy of patients was very low. Applying this measurement to the patients of group B allowed the therapeutic decision of port system decompression. The only patient from this group, who died, had a splenorenal shunt. He died 11 months after the intervention, in the context of continuing the consumption of ethanol.

## Conclusion

The measuring of HVPG allowed the selection of a high-risk group of patients.

This allowed the making of a therapeutical decision with a significant prolongation of life in these patients (34.2 vs. 15.5 months).
